# Correction to: Effects of exergaming on cardiovascular risk factors and adipokine levels in women

**DOI:** 10.1007/s12576-017-0588-y

**Published:** 2018-01-05

**Authors:** Maria Guadalupe Soares Amorim, Maurício Dias de Oliveira, Daiane Santos Soares, Leandro da Silva Borges, Alexandre Dermargos, Elaine Hatanaka

**Affiliations:** 1 Instituto de Ciências da Atividade Física e Esportes, Universidade Cruzeiro do Sul, Rua Galvão Bueno, 868, 13° Andar, Bloco B, Liberdade, São Paulo, SP 01506-000 Brazil; 2 Universidade Paulista (UNIP), São Paulo, SP Brazil

## Correction to: The Journal of Physiological Sciences 10.1007/s12576-017-0581-5

The article “Effects of exergaming on cardiovascular risk factors and adipokine levels in women”, written by Maria Guadalupe Soares Amorim, Maurício Dias de Oliveira, Daiane Santos Soares, Leandro da Silva Borges, Alexandre Dermargos and Elaine Hatanaka, was originally published electronically on the publisher’s internet portal (currently SpringerLink) on 30 November 2017 without open access.

With the author(s)’ decision to opt for Open Choice the copyright of the article changed on [date the updated version will be/was published] to © The Author(s) 2017 and the article is forthwith distributed under the terms of the Creative Commons Attribution 4.0 International License (http://creativecommons.org/licenses/by/4.0/), which permits use, duplication, adaptation, distribution and reproduction in any medium or format, as long as you give appropriate credit to the original author(s) and the source, provide a link to the Creative Commons license and indicate if changes were made.

The original article was corrected.

